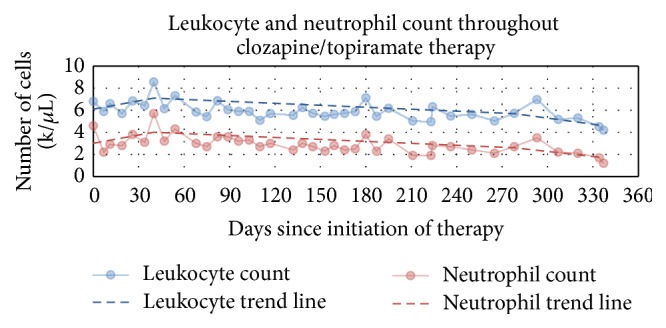# Concomitant Use of Topiramate Inducing Neutropenia in a Schizophrenic Male Stabilized on Clozapine

**DOI:** 10.1155/2016/6086839

**Published:** 2016-01-24

**Authors:** Pravesh Sharma, Jeffrey Davis, Vivekananda Rachamallu, Manish Aligeti

**Affiliations:** Department of Psychiatry, Texas Tech University Health Sciences Center, 3601 4th Street, Stop 8103, Lubbock, TX 79430, USA

## Abstract

This is a case of a 23-year-old African American male with a history of paranoid schizophrenia that developed neutropenia on a clozapine-topiramate therapy. Clozapine had well addressed the patient's psychotic symptoms, while topiramate was used as a weight-lowering agent. The patient had fairly stable leukocyte counts for eight months on clozapine 300 mg and topiramate 100 mg daily. Doubling the dosage of topiramate led to severe neutropenia after two months. Reviewing the patient's laboratory reports showed a gradual decline of neutrophils occurring at a lower dosage, followed by a rapid decline after an increased dosage. In this case, we report that not only did topiramate act as the neutropenic agent, but also it might have done so in a dose-dependent manner.

## 1. Introduction

Atypical antipsychotics have been preferred over first-generation or typical antipsychotics due to their decreased extrapyramidal side effects [1]. However, they are also associated with metabolic syndrome, particularly weight gain, and clozapine has been shown to have the greatest impact on weight gain among all atypical antipsychotics [2]. A common method of countering this effect is treatment with topiramate [3]. In a 12-week naturalistic, open study to understand the benefits of topiramate in individuals suffering from schizophrenia and treated with clozapine, topiramate augmentation led to a 2.5% decrease in body weight (*P* = 0.015). A meta-analysis of nine studies examining its use in nonpsychiatric patients for treatment of obesity found a 6.5% rate of weight loss at 6 months [4]. Clozapine has also been well known to cause leukopenia, particularly neutropenia. Regular laboratory interventions are mandated when a patient is started on clozapine as per guidelines [5]. Typically, topiramate is not considered a neutropenic agent, but the effects of cotherapy on decrease in leukocytes have not been thoroughly studied or reported.

## 2. Case Report

Mr. S, a 23-year-old African American male with the diagnosis of paranoid schizophrenia, was hospitalized in our inpatient psychiatric hospital approximately 15 months ago. The patient had failed trials of several typical and atypical antipsychotics. Leading up to the admission, he was having religious delusions, ideas of reference, and thoughts of cutting his arm (he had lacerated his arm prior to that admission). He was displaying thought blocking and poverty of speech. His laboratory data, including complete blood count, basal metabolic profile, and thyroid function tests, were within normal limits. His urine drug screen was negative except for cannabinoids and blood alcohol level was less than 5 milligram per deciliter. Based on that, he was started on clozapine. He was discharged with a dosage of 200 mg of clozapine daily and was instructed to return to outpatient psychiatry clinic in a week. During that appointment, the patient continued to exhibit psychotic symptoms and also had gained considerable weight (345.7 pounds, body mass index of 42.7 kg/m^2^). Subsequently, clozapine was increased to 250 mg per day for one week and to 300 mg per day thereafter. In addition, topiramate was initiated at 25 mg per day for one week and 50 mg per day for the following two weeks and then finally increased to 100 mg per day until the following appointment. Mr. S continued clozapine 300 mg daily and topiramate 100 mg daily for the following eight months, during which his WBC and ANC were within the normal range. After eight months on this regimen, his weight continued to increase (370.2 pounds, body mass index of 48.28 kg/m^2^) but clinically he was doing well and he denied symptoms of psychosis and reported euthymic mood. In lieu of this, topiramate was increased to 200 mg per day, with no change in clozapine dosage. After 13 days, it caused a temporary spike in WBC to 7,000/*μ*L and neutrophils to 3,500/*μ*L, following which it showed an abrupt decline in leukocyte (neutrophil) count: 5,200/*μ*L (2,200/*μ*L) on the 27th day, 5300/*μ*L (2,100/*μ*L) on the 40th day, and 4,500/*μ*L (1,700/*μ*L) on the 54th day. On the 57th day, the pharmacy denied a refill for clozapine due to the lowered neutrophils. The patient's ANC count further plummeted to 1200/*μ*L, while WBC count was 4,200/*μ*L. A day later, the patient's laboratory results showed 5,200 WBC/*μ*L and 1,900 neutrophils/*μ*L (Figure 1). Considering that and assuming that clozapine was the cause of agranulocytosis, he was admitted to our psychiatric inpatient facility for medication management and monitoring of laboratory measurements. The patient was not experiencing any significant psychotic symptoms at that time.

At the time of admission, the patient had not been taking clozapine for two days as the pharmacy refused to refill it. During the hospital course, topiramate was tapered off, the patient was started on olanzapine 10 mg daily, and lithium was started to aid in mood stabilization and to help control WBC/ANC levels by virtue of its ability to potentially cause leukocytosis [6]. The patient tolerated the transition from clozapine to olanzapine and lithium. He denied hearing voices and other symptoms of psychosis. After starting lithium, WBC spiked to 7,800/*μ*L and ANC to 4,800 *μ*L. At time of discharge, Mr. S had 5,800 WBC/*μ*L and 2,600 neutrophils/*μ*L, with similar values two days later at outpatient follow-up.

## 3. Discussion

A review of the patient's medical records was focused on WBC and ANC values in relation to medication dosing and changes. A literature search was also performed using the PubMed database. The parameters of the search included all English language publications (excluding review articles) between 1990 and 2015 which contained the words topiramate or topiramate in combination with the words leukopenia, neutropenia, or agranulocytosis. This returned a total of three results, including two case reports describing topiramate-induced leukopenia or neutropenia, only one of which involved concurrent clozapine therapy.

There is empirical evidence of leukopenia or neutropenia resulting from clozapine and from a combination of clozapine and other psychotropic drugs [7, 8], but a search of topiramate-induced leukopenia or neutropenia is scarce. The search produced two reported cases. In one case a patient with a two-year history of being on clozapine developed leukopenia after the addition of topiramate due to concerns about weight gain. Consequently, topiramate was stopped while clozapine was restarted. The patient's leukocyte count then returned to the normal limits [9].

A second case report described a patient with intractable partial epilepsy who developed agranulocytosis after topiramate was added to a phenytoin and acetazolamide regimen. Topiramate was decreased and eventually discontinued causing WBC to return to the normal limits [10].

Our case suggests a dose-dependent effect of topiramate when used with another leukopenic agent. Mr. S showed an upward trend in WBC and ANC during the 6 weeks prior to reaching the combination of clozapine 300 mg and topiramate 100 mg. At these doses, WBC and ANC levels gradually declined and then decreased more rapidly after topiramate was increased to 200 mg. Clozapine is mostly metabolized by CYPIA2. However, drugs that inhibit CYP2D6 are reported to elevate clozapine plasma level. There is little data about the interaction between topiramate and antipsychotic drugs. It is worth noting that topiramate has no effect on CYPIA2 and CYP2D6, which are the main metabolizing enzymes of clozapine [11].

## 4. Conclusion

Care should be taken when considering adding topiramate concurrently with any neutropenic agent. While the benefits of weight control may be desired, patients and their families should be counseled that the addition of topiramate to clozapine might cause decreased leukocytes or neutrophils, even if the patient has not previously had neutropenia while on clozapine. Nevertheless, if topiramate is started, the dose should be increased slowly and laboratory values monitored carefully. As established in our case report, this may be a dose-dependent effect. Dosage changes should be spaced apart enough to monitor the trends in leukocyte counts and special care should be taken when considering an increase of topiramate to 200 mg daily. An increase in frequency of complete blood count tests might be considered to provide closer attention to and changes in trends.

## Figures and Tables

**Figure 1:**